# The Manipulative Complexity of Lower Paleolithic Stone Toolmaking

**DOI:** 10.1371/journal.pone.0013718

**Published:** 2010-11-03

**Authors:** Aldo Faisal, Dietrich Stout, Jan Apel, Bruce Bradley

**Affiliations:** 1 Department of Bioengineering and Department of Computing, Imperial College London, London, United Kingdom; 2 Department of Anthropology, Emory University, Atlanta, Georgia, United States of America; 3 Department of Archaeology and Osteology, Gotland University College, Visby, Sweden; 4 Department of Archaeology, Exeter University, Exeter, United Kingdom; University of Oxford, United Kingdom

## Abstract

**Background:**

Early stone tools provide direct evidence of human cognitive and behavioral evolution that is otherwise unavailable. Proper interpretation of these data requires a robust interpretive framework linking archaeological evidence to specific behavioral and cognitive actions.

**Methodology/Principal Findings:**

Here we employ a data glove to record manual joint angles in a modern experimental toolmaker (the 4^th^ author) replicating ancient tool forms in order to characterize and compare the manipulative complexity of two major Lower Paleolithic technologies (Oldowan and Acheulean). To this end we used a principled and general measure of behavioral complexity based on the statistics of joint movements.

**Conclusions/Significance:**

This allowed us to confirm that previously observed differences in brain activation associated with Oldowan versus Acheulean technologies reflect higher-level behavior organization rather than lower-level differences in manipulative complexity. This conclusion is consistent with a scenario in which the earliest stages of human technological evolution depended on novel perceptual-motor capacities (such as the control of joint stiffness) whereas later developments increasingly relied on enhanced mechanisms for cognitive control. This further suggests possible links between toolmaking and language evolution.

## Introduction

Lower Paleolithic stone tools provide under-utilized evidence of the timing and context of human cognitive evolution that is not available from comparative studies. Of particular interest is the relative contribution of perceptual-motor vs. cognitive adaptations at various points in human technological evolution [e.g. 1], [2], [3], [4]. Increasingly complex prehistoric toolmaking methods through time offer insight into evolving capacities but require a robust interpretive framework linking archaeological evidence to specific behavioral and cognitive actions. We recorded the joint and abduction angles of the hand digits of a modern experimental toolmaker (the 4th author) replicating ancient stone tools in order to better characterize the manipulative complexity of two major Lower Paleolithic technologies, and to compare this with recent functional brain imaging studies of the neural bases of these same technologies [5], [6].

**Figure 1:**
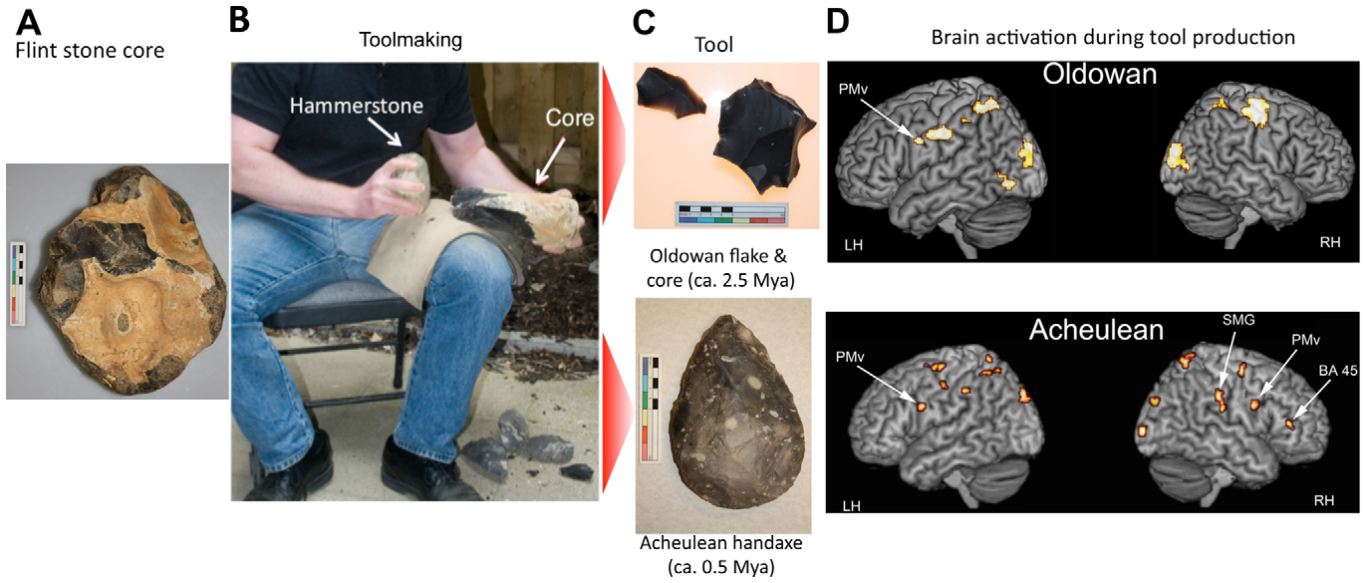
Lower Palaeolithic toolmaking. A stone ‘core’ (A) is struck with a hammerstone (B) in order to detach sharp stone ‘flakes’. In Oldowan toolmaking (C, top) the detached flakes (left in photo) are used as simple cutting tools and the core (right in photo) is waste. In Acheulean toolmaking (C, bottom), strategic flake detachments are used to shape the core into a desired form, such as a handaxe. Both forms of toolmaking are associated with activation of left ventral premotor cortex (PMv), Acheulean toolmaking activates additional regions in the right hemisphere, including the supramarginal gyrus (SMG) of the inferior parietal lobule, right PMv, and the right hemisphere homolog of anterior Broca's area: Brodmann area 45 (BA 45) (Imaging data adapted from [6]).

The technologies studied were Oldowan flake production and Late Acheulean handaxe making Figure 1A–C), representing the beginning and end of the Lower Paleolithic. The Lower Paleolithic itself encompasses some 90% of human prehistory, beginning with the first stone tools 2.6 million years ago (mya) and lasting more than 2 million years. The earliest known tools are assigned to the Oldowan Industry and consist simply of sharp stone flakes struck from river cobbles through direct percussion with another stone [7], [8]. Nevertheless, their production involves considerable perceptual-motor skill [5], [9]. After about 1.7 mya [10], intentionally shaped Acheulean tools began to appear, including large, teardrop-shaped cutting tools known as ‘handaxes’ (Figure 1). By the Late Acheulean some 0.5 mya, these forms had achieved a high level of refinement and standardization, reflecting increasingly elaborate and skill-intensive shaping techniques [10].

The development of intentional shaping in the Acheulean has been considered a key event in human cognitive evolution, reflecting new capacities for the “imposition of arbitrary form” [11] and the presence of more complex mental [12] and/or procedural “templates” [13]. Such observations have led various researchers to consider possible links between stone toolmaking and language evolution [e.g. 11], [14], [15]. More recently, functional brain imaging studies of experimental Oldowan [5] and Acheulean [6] toolmaking have provided direct evidence of neural overlap between language and toolmaking in inferior frontal cortex (Figure 1D). Oldowan toolmaking was associated with activation of left ventral premotor cortex, a region known to be involved in both manual grip coordination [16], [17] and phonological processing [18], [19], [20]. Acheulean toolmaking differed from Oldowan in producing additional activity in the right hemisphere (Figure 1D), including the supramarginal gyrus of the inferior parietal lobule, the right ventral premotor cortex, and the right hemisphere homolog of anterior Broca's area (Brodmann area [BA] 45) [6], a region that is bilaterally involved in higher-order hierarchical cognition [21] and which is specifically implicated in the processing of linguistic context and prosody (intonational contours) [19].

What these experiments did not make clear was whether increased right hemisphere activation reflected increased demands for grasp control in the contralateral hand, distinctive right hemisphere contributions to the cognitive control of complex action sequences [22], [23], or both. During Paleolithic toolmaking (Figure 1A–C), the non-dominant hand plays a critical role supporting and orienting the stone ‘core’ from which flakes are detached by relatively invariant ballistic strikes from a ‘hammerstone’ held in the dominant hand. We wanted to further investigate the role of the non-dominant hand in Oldowan and Acheulean technologies in order to clarify its relationship with the observed contralateral brain activation.

**Figure 2:**
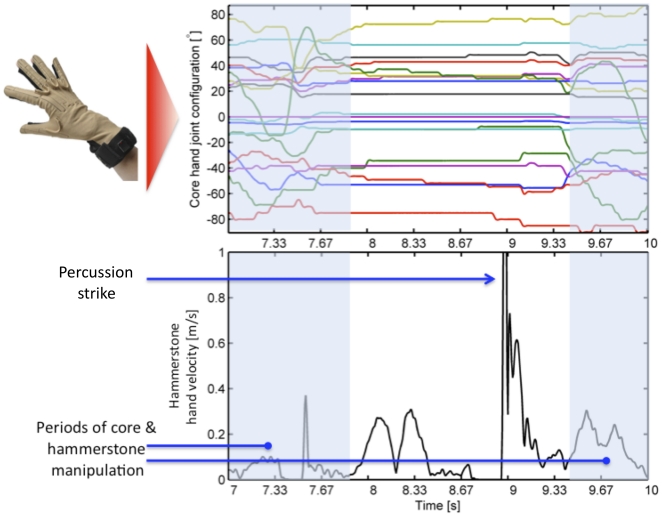
Stability of the core-holding (left hand) and velocity profile of the hammerstone wielding hand (right hand), as well as manual video annotation of a ‘percussion’ event (Top). Time series of the 18 joint sensors on the data glove (photo left). Period of rapid hand configuration changes (blue shaded region) frame a period of stability (clear region). This stability period corresponds to the period just before and during a percussion strike, as obtained from the manually annotated video. (Bottom) Velocity profile of the hammerstone wielding hand aligned with the plot above. The ‘percussion’ event is clearly visible in the velocity spike (top blue arrow).

**Figure 3:**
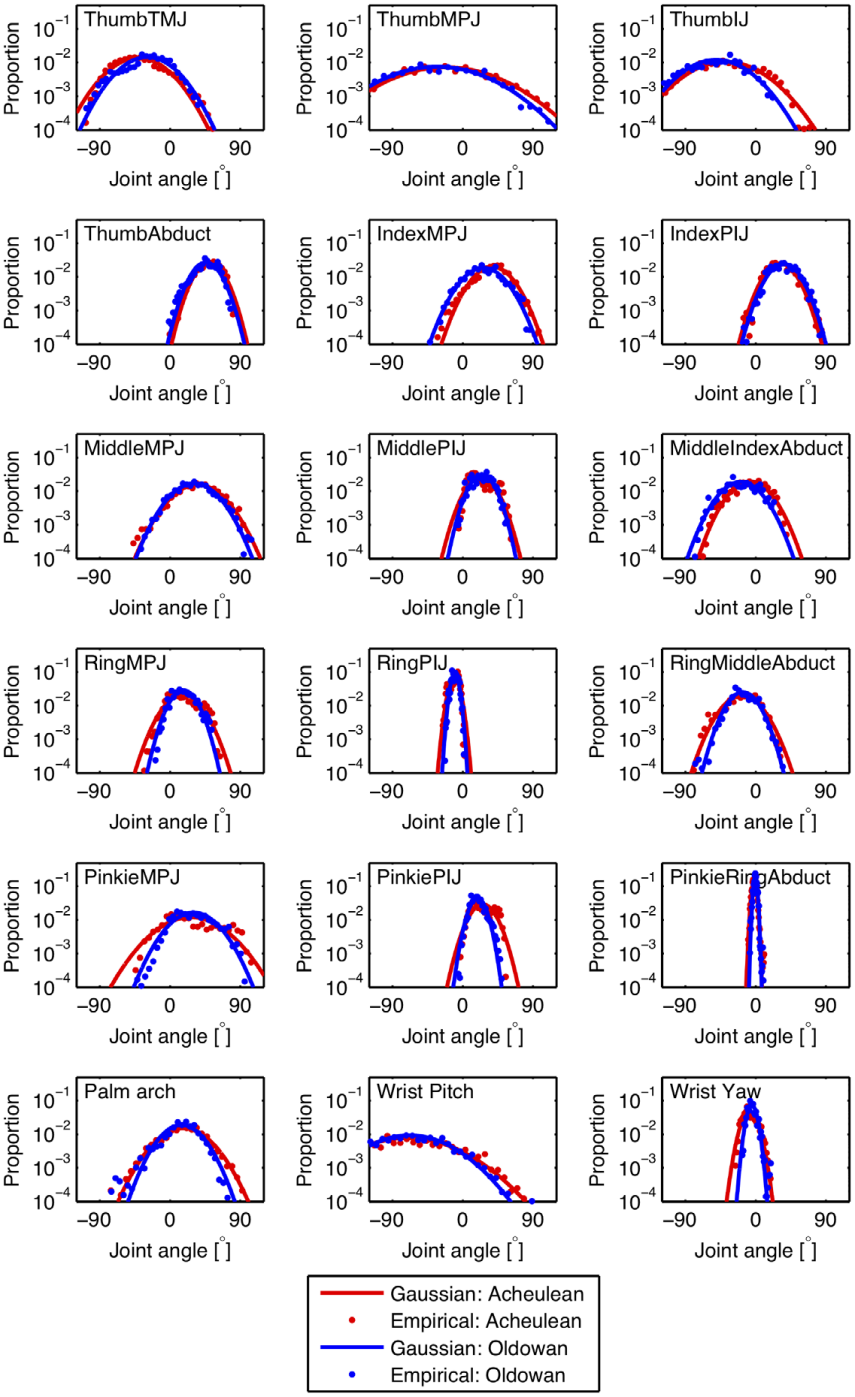
Probability distribution (or relative frequency or proportion of total time) of joint angles for 18 joint angles of the left hand during Oldowan (blue dots) and Acheulean (red dots) toolmaking. Solid lines are Gaussian distributions with mean and standard deviation matched to the empirical joint angle histograms. Plots have a logarithmic vertical axis, such that the data and the matching Gaussian distributions appear as parabolas. The configuration of the hand was determined by the following joints (from left to right, top to bottom): the carpo-metacarpal (ThumbTMJ), metacarpal-phalangeal (ThumbMPJ) and interphalangeal (ThumbIJ) joint angles for the thumb and the abduction angle (ThumbAbduct) between the thumb and the palm of the hand, the metacarpal-phalangeal (MPJ) and proximal interphalangeal (PIJ) joint angles for the four fingers, the three relative abduction angles between the four fingers (MiddleIndexAbduct, RingMiddleAbduct, PinkieRingAbduct), as well as the arching and bending (during flexion/extension, ulnar/radial deviation) of the palm surface with respect to the wrist (PalmArch, WristPitch,WristYaw). Note, the joint angle zero is relative to the joint angle on our calibration splints.

**Figure 4:**
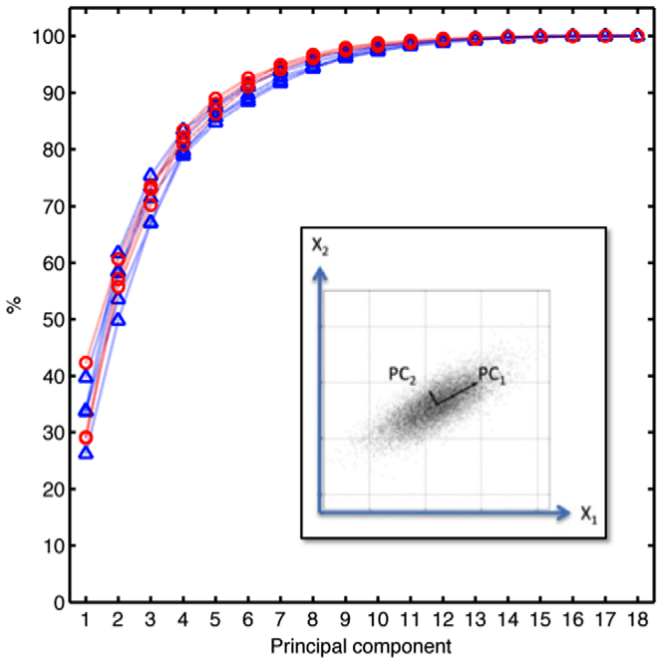
Variability explained by principal components of stable hand configurations (data from automatic annotation). Curves show the cumulative sum of variance explained by increasing numbers of principal components for 5 Acheulean and 3 Oldowan reduction sequences. Principal component analysis is of Acheulean (blue triangles, one for each of the corresponding 5 toolmaking sequences and for each principal component) and Oldowan (red circles, 3 toolmaking sequences) hand configuration data of the core holding (left) hand, when the hammerstone hand was moving faster than 0.5 m/s. Note, that some data points overlap for Principal Component 1 and higher order Principal Components. Inset: Conceptual drawing of Principle Component Analysis (PCA). PCA is a linear transformation that seeks to explain multiple, correlated dimensions (X1, X2) of variation in the data (grey cloud) in terms of uncorrelated dimensions termed principal components (PC1, PC2). This linearly uncorrelated representation can then be used to reduce the dimensionality of the 2-dimensional data, e.g. describing the data set by using only PC1 as 1-dimensional data set (effectively capturing the longitudinal characteristic structure of the data). We do not reduce the dimensionality of the data *per se*, but use the relative amount of variance explained by each principle component as a characteristic value for the complexity of the data set.

**Figure 5:**
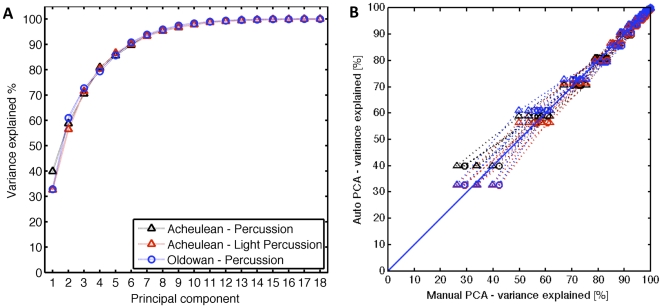
(A) Cumulative variability (%) explained by principal components of stable hand configurations (from manual annotation). Data points overlap for Principal Component(PC) 1 and higher order PCs. (B) Comparison of manually versus automatically annotated data. Plot of variance explained by principal components analysis of stable hand configurations: Variance explained of all principal component calculated for manually classified Acheulean percussion events (blue), Acheulean light percussion events (black) and Oldowan percussion events (red) plotted versus corresponding principal components of automatically annotated Acheulean (triangle) and Oldowan (circle) percussion-like events. E.g. the curve with black triangles, shows the principal components from 1 to 18 of the Acheulean light percussion events extracted by manual annotation (as horizontal position of the black triangle) versus the corresponding principal components from 1 to 18 for the automatically extracted percussion events (as vertical position of the black triangle). Note: automatic annotation did not distinguish between light percussion and percussion events. Correlation coefficients were greater 0.95 for all curves confirms that manual and automatic annotation yield very similar results.

Previous experimental studies of stone toolmaking have provided insight into the manual grips [24], [25], [26], elementary movements [27], [28], [29] and kinematic synergies [30] involved in this complex perceptual-motor skill. To date, however, such studies have either been based on qualitative grip typologies (as characterized ‘by-eye’) or been confined to the movements of the striking arm proximally from the wrist. We employed a data glove to record digit joint angles in the left, core-holding hand of an expert right-handed toolmaker during Oldowan flake production and Late Acheulean handaxe shaping. The high precision recording of manual joint angles enabled us to quantify grip diversity and complexity in an objective, mathematically principled manner (Figures 2, 3, 4, 5). This allowed us to directly compare the manipulative complexity of Oldowan and Acheulean technologies.

**Figure 6:**
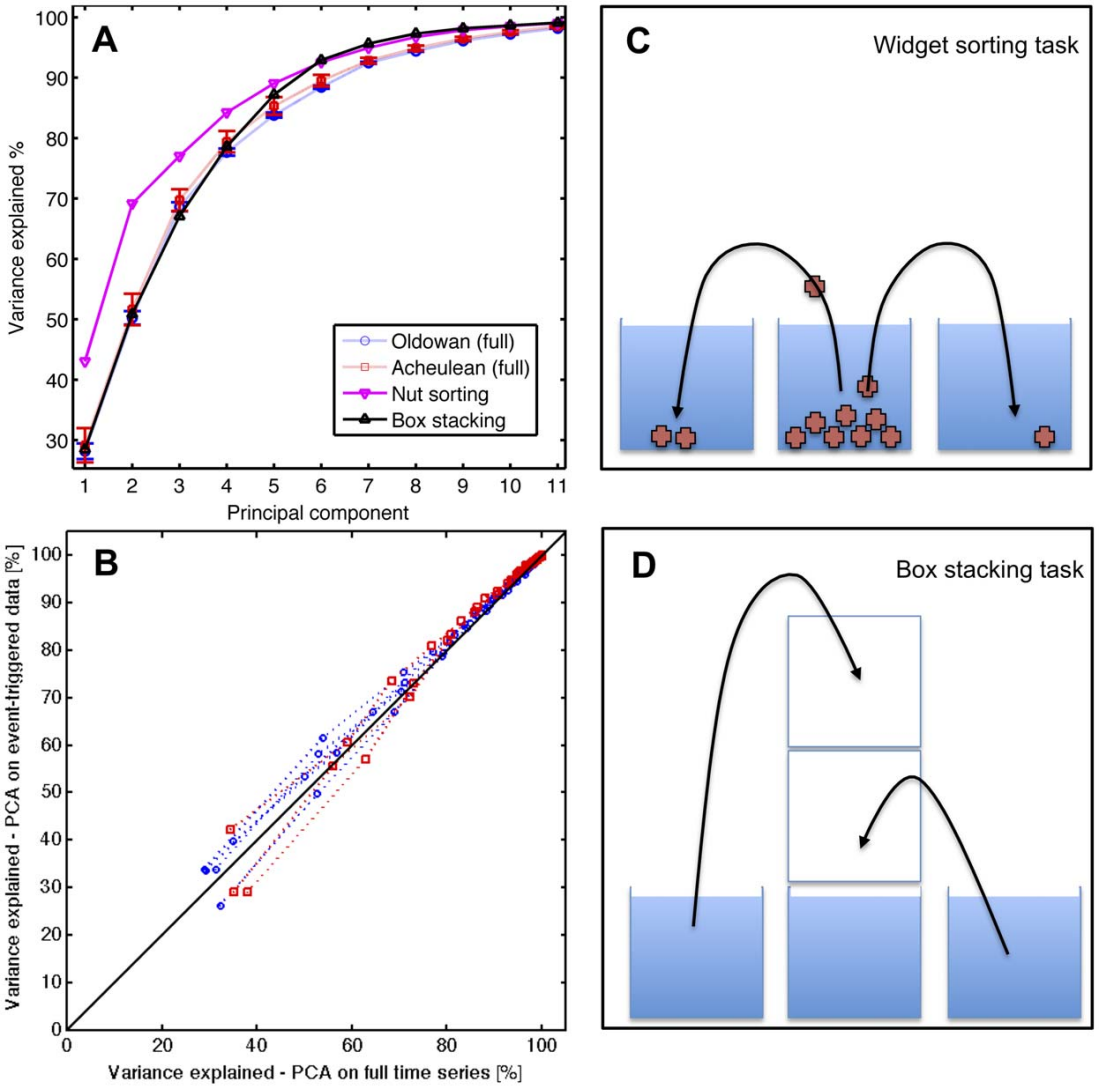
(A) Cumulative variability explained by principle components (from the full set of the hand configuration time series data) for toolmaking (Oldowan - circle, Acheulean - square) and control tasks (widget sorting - downward pointing triangle, box stacking - upward pointing triangle). Small object manipulation is clearly less complex than toolmaking (greater variance explained by PC 1 and above). Box stacking is much more comparable, but is still distinguished from PCs 6, 7,8 and onwards. (B) Comparison of using the full time series versus the event-triggered (percussion events) to calculate the PCA variance explained demonstrate that they are very similar. Blue circles are the corresponding variance explained of the PCA extracted from Oldowan trials (red squares Acheulean trials). (C,D) Schematic of the object manipulations performed in the control task. (C) individual nut-sized widgets were picked from a box and placed in alternating adjacent boxes. (D) Boxes (styrofoam, top open) were stacked upon each other and then unstacked again.

To confirm the capacity of this novel method to differentiate manual activities, we collected additional data from two control tasks (Figure 6), both involving manipulative actions of the left hand. In the first task, small nut-sized objects (“widgets”) were grasped and transferred between containers. This involved the use of a 4-digit pad-to-pad grip, and loosely approximates the kind of small object manipulation involved in primate manual foraging. Functionally, it is analogous to the unimanual grasping and positioning of nuts on an anvil during chimpanzee nut-cracking, although the manual anatomy and preferred grips clearly differ between species. In the second task, Styrofoam boxes were repeatedly stacked and un-stacked in a carefully aligned column. These open boxes afford a wide array of different grasps during manipulation, and provide an example of structured interaction with complex artifacts in the modern built environment.

## Results

To systematically quantify the grips we used a CyberGlove I data glove that recorded the angles of the joints of each digit and the abduction angles between digits. In total, the glove data has 18 sensors, spanning an 18-dimensional space of joint angles for the hand. We found that throughout the toolmaking process of both Oldowan and Acheulean techniques the proportion of time the individual joints spend in specific configurations was similar and displayed a Gaussian (“Normal”) distribution (Figure 3).

A major challenge in the quantitative analysis of behaviour is its variability [31]. This is clearly visible in the histograms of joint angles during Oldowan and Acheulean toolmaking (Figure 3). For this study, we had to consider that each toolmaking sequence has unique, uncontrollable elements, such as the shape and internal properties of the core, which are gradually revealed during the toolmaking process. The grips would thus differ in detail between cores, as well as gradually changing for a single core as flake removal progressively alters core form. Furthermore, we might expect systematic differences in variability across different toolmaking methods. A simple count of qualitatively defined grip types would thus provide insufficient information to evaluate the true manipulative complexity of this real-world activity.

We instead took a computational-statistical view of motor behaviour to produce a quantitative estimate of the complexity of hand configurations. Previous studies of human motor control show that normal hand behavior uses only a small subset of the possible hand configurations [32] and that we can extract these using dimensionality reduction techniques. Dimensionality reduction techniques can be illustrated by considering the index finger, which has 3 joints controlled by 5 muscles. Describing the flexing behavior of this finger requires *a priori* 3 values (“dimensions”). However, in specific movements like making a fist, as we flex one joint of the index finger we flex the other two joints in a highly coordinated manner. Thus, we would require, in principle, a single value to describe the configuration of the full finger. If this were systematically the case for all our movements, we would just require 1 dimension to describe the configuration of the finger. In reality this dimensionality varies somewhere between 1 and 3, depending on the amount of coordination and correlation of the hand's joints in our movements and actions. This is what we systematically measured for the actions of tool making using the data glove.

We used Principal Component Analysis (PCA) as dimensionality reduction technique, which is well suited given the Gaussian distribution of the joint statistics (Figure 3). PCA reduces a set of correlated variables, the joint angles of the hand, into a set of uncorrelated variables called principal components (Figure 4, inset). The first principal component accounts for as much of the variability (as quantified by the variance) in the data as possible, as does each succeeding component for the remaining variability. We therefore use PCA as a measure for the complexity of hand configurations, by measuring how many principal components can explain how much variability in the data. For example, a simple behavior, e.g. curling and uncurling a hand into a fist, would reveal a single dominant principal component as all 5 fingers (and each finger's joint) move in a highly correlated manner. In contrast, a complex behavior, such as expert typing on a keyboard would reflect five dominant principal components as each finger moves independently from the others. In fact, a previous study that measured human finger movements (i.e. joint angle velocities) over several hours of normal modern-day activity showed that about 75% of the variance in the data could be explained by the first four principal components [31].

From toolmaking experience and basic biomechanics, we assume that grip stiffness of the hand holding the core (left hand) should peak around the time of hammerstone impact (Figure 2). Using a simple automatic annotation method, we performed principal component analysis of the hand configuration during high velocity impact events of the (right) hammerstone-wielding hand. Plotting the variance explained versus principal components shows (Figure 4), that across the five Acheulean and three Oldowan sequences, the first principal component explained 25–40% of the variance in the data. The first two principal components explain about 50–62.5% of the variance. About 85% percent of the data can be explained by the first five principal components. Moreover, for each principal component, there is no significant difference between the Acheulean and Oldowan variance explained. We tested this by conducting a t-test for each principal component, assuming that the population of values for that principal component from both Oldowan and Acheulean data were drawn from a distribution with the same mean (DF = 7, p<0.05 for all 18 principal components).

To further corroborate our findings, we manually annotated video recordings of one Acheulean and one Oldowan tool production episode. Eight different technical actions, including striking actions, were recorded with sub-second precision. We performed principal component analysis for the set of hand configurations on which instantaneous ‘percussion’ events (fast, hammer-strike like motions) or ‘light percussion’ events (fast, chiseling-like motions) were annotated in the video. ‘Light percussion’ is a form of edge modification used to facilitate the removal of special ‘thinning’ flakes in Acheulean toolmaking and is absent in Oldowan toolmaking. Plotting the variance explained versus the number of principal components for Acheulean and Oldowan toolmaking produced very similar results (Figure 5A). The first principal component explains 30–40% of the variance; the first and second principal components explain 55–60%. As in the automatically annotated case, about 85% of the variance is explained by the first five principal components.

Comparing the manual and the automated data directly corroborates these findings (Figure 5B). The principal components between the two methods of tagging the data are highly correlated, as can be seen by plotting the variance explained from Acheulean ‘percussion’ (blue data), Acheulean ‘light percussion’ (black data), and Oldowan ‘percussion’ (red data) from manual annotation data against variance explained from automatically annotated data from striking events in Acheulean (triangles) or Oldowan (circles) (all pairwise correlation coefficients had ρ = 0.95). Thus, the results indicate that the structure of non-dominant (i.e. core manipulation) hand configurations for Acheulean vs. Oldowan percussions is equally complex.

In addition to these findings and two confirm the validity of our behavioral complexity measure, we performed two control experiments involving everyday object manipulation tasks: small object (“widget”) sorting and box stacking (Figure 6). We wanted to compare the manipulative complexity of toolmaking with its characteristic percussion events to these rather differently structured control tasks. Therefore, to remove any bias we analysed the full hand configuration time series of the toolmaking sequence (and not just the subset of stable hand configurations) and compared these to the full time series of hand configurations of the two control tasks. Results show that both control tasks have lower complexity than stone toolmaking (Figure 6A). Widget sorting was considerably less complex than the other 3 manipulation tasks, with the first 2 principal components capturing 70% variance as compared to 50–53% in toolmaking and box stacking. The box stacking task was somewhat more complex, and closely approximates stone toolmaking across the first 4 principle components. However, the lesser manipulative complexity of box stacking is evident in the 5^th^ and 6^th^ components which explain 5–10% more variance than in either Oldowan or Acheulean toolmaking. Finally, we found that stone toolmaking complexity as measured from the full time series and from the percussion-triggered events was very similar (Figure 6B).

This series of results confirms that the manipulative complexity of Oldowan and Acheulean toolmaking are indistinguishable. Furthermore, we showed that complexity measures for stable hand configurations during toolmaking and for the full time series including manipulation between stable grips are very similar. Finally, we demonstrated that toolmaking complexity is clearly higher than the much simpler sorting task of nut-size “widgets” and box stacking tasks that we used as controls.

## Discussion

We used a data glove and electromagnetic position markers to quantify the modern day reproduction of Lower Paleolithic stone tools. To this end we used a novel, principled, and general measure of behavioral complexity based on the statistics of movements occurring during the task. The application of this general technique to the specific investigation of the complexity and diversity of left hand grips required to produce Acheulean and Oldowan artifacts allowed us to make comparisons with previously observed patterns of lateralized brain activation during stone toolmaking (Figure 1). Increasing anatomical and functional asymmetry is a key trend in human brain evolution, and apparent left hemisphere dominance for both language and manual praxis has inspired influential hypotheses linking human handedness, tool use and language [e.g. 33]. Activation of left ventral premotor cortex, a region also involved in phonological processing [20], during Oldowan toolmaking is consistent with these ideas and suggests that early stone toolmaking (ca. 2.6 mya) could have contributed to the evolution of neural substrates also important for articulate speech [34]. However, the increased right hemisphere activity seen during Acheulean toolmaking is unexpected in this framework.

An alternative framework [35], [36] emphasizes the complementary roles of left and right hands in everyday human manipulative behaviors (e.g. cutting bread, hammering a nail, writing on paper, striking a match, washing dishes), in which the left hand typically provides a stable postural support for the higher frequency actions of the right hand. This may correspond to a similar hemispheric “division of labor” in the brain [6], with left hemisphere preferentially involved with rapid, small-scale processing and right hemisphere with larger-scale, longer-duration processing [37]. Indeed, it is becoming more widely appreciated that right hemisphere plays a critical role in larger-scale prosodic and contextual aspects of language processing [19], [38] and in the coordination of multi-step manual action sequences [22], [39]. In this framework, right hemisphere involvement in Acheulean toolmaking suggests the presence of additional cognitive demands for behavioral integration over time and the possibility of evolutionary interactions between (Late) Acheulean (ca. 0.5 mya) toolmaking and discourse-level language processing [34].

A major challenge to such interpretations, however, is the possibility that increased right hemisphere activity during Acheulean toolmaking simply reflects an increased diversity/complexity of left hand grips, without necessarily implicating distinctive right hemisphere processing characteristics. In this case, activation of right BA 45 would still imply increased demands for the hierarchical organization [21] of (left hand) actions, however the observed lateralization could simply be attributed to the localization of cognitive control to the same hemisphere as task execution [40]. In order to test this hypothesis it was necessary to quantify the diversity/complexity of left hand grips deployed during Oldowan and Acheulean toolmaking in a principled, quantitative manner allowing for direct comparison.

This is not a trivial undertaking because the details of the required grips vary inherently as core shapes differ. Moreover, for each core, the specific grip will have to vary during the transformation of the core into a tool. The challenge was to discover an underlying and quantitative simplicity which accounts for and does not neglect variability in behavioral data, thus taking a view we refer to as Bioinformatics of Behavior [41]. We used a statistical measure to quantify the complexity of grips, Principal Component Analysis, which ignores individual differences in the grips but looks for a common structure to all grips in a data set. Our approach to quantify behavioural complexity is not restricted to hand joint angles, but can be applied to other behavioural time series, such as full body joint movements or animal data.

Our behavioral complexity measure allowed us to compare structure across all stable grips in Acheulean vs. Oldowan toolmaking. We quantified this, and found little difference in the number of principal components required to explain a given amount of variability in the stable hand configurations, indicating an equally complex structure. Moreover, the hand configuration variance explained per principal component provides a measure of the breadth of the statistical distribution of hand configurations. Thus the variance explained reflects the diversity of grips, which again overlaps between Acheulean and Oldowan toolmaking.

Interestingly, grip complexity in Lower Paleolithic toolmaking, as quantified by variance explained per principal component, is broadly comparable to modern-day daily manipulative complexity measured with the same data glove technology (cf. data plotted Figure 3.A in [32]). Although this previous study considered dynamic hand movements rather than stable hand configurations, the variance explained by a given number of principle components in each case is typically within 5–10% of the other. The most notable divergence is the lower percentage of variance (∼10% less) explained by the first two principle components in stone toolmaking, which might suggest the presence of distinctive manipulative patterns as compared to modern daily activities. However, the tasks performed during the daily activity study were unknown and the capacity of this method to distinguish more and less complex tasks has yet to be directly demonstrated. To this end we measured, for the first time, the manipulative complexity of specific real-world tasks: small object sorting and box stacking. We expected small object sorting to show a much lower complexity than core manipulations, as it only involves picking up and transferring identical objects – quite unlike manipulating a constantly changing core during toolmaking. Similarly, we expected the box stacking task would require an intermediate complexity between small object sorting and core manipulation, because more grasp variety and fine control is needed than in the sorting task, yet the shape of the objects being manipulated does not change as in stone toolmaking. These expectations were corroborated by our results, showing both control manipulations to be less complex than the two toolmaking tasks.

Having validated the method, the absence of differences in left hand grip complexity between Oldowan and Late Acheulean toolmaking remains a surprising result, especially considering the substantial differences between the two technologies. Both require that the core be properly positioned and firmly supported, yet Acheulean toolmaking involves both a greater diversity of technical actions and more substantial changes in core morphology as the core is shaped into a pointed, thin and symmetrical ‘handaxe’. Furthermore, as the piece is thinned it becomes increasingly important to properly brace it to prevent breakage, a problem that is not present in Oldowan toolmaking. Nevertheless, results indicate that the degree of grip complexity and diversity already present in Oldowan toolmaking is sufficient to accommodate Later Acheulean toolmaking.

Stable hand configurations are essential for skilled tool making. Recent work in human motor control has uncovered how finely the nervous system controls the stability of mechanically unstable objects during tool manipulation (e.g. when applying a screw driver, or in our case when the hammerstone hits the edge of the core). This is achieved through the orchestrated co-contraction of antagonistic muscles so as to stiffen a joint against undesired perturbation [42]. Humans can tune the stiffness of joints (also known as mechanical impedance) to optimally compensate for internal or external perturbations, trading off the degree of muscle co-contraction (which is metabolically expensive and promotes muscle fatigue) with the required mechanical stability of a task [43], [44]. Thus, the stable hand configurations we observed in toolmaking may arise due to a need to minimize the effects of internal perturbations, e.g. maintaining the precise orientation and location of the core's edge for the percussion motions of the other arm, and external perturbations, e.g. direction and force of the hammerstone impact.

Experimental data shows that the stiffness of the fingertips depends on the specific configuration of the hand [45]. This may suggest the existence of a specific subset of hand configurations in which stability of the core prior to and during striking is maximized. A recent study on how the nervous system controls force sharing in the fingers for object grasping and pickup, studied an object that had a varying center of mass on each trial (akin to the changing center of mass of the core as it is reduced) and showed a stereotyped pattern of force production across the fingers of the non-dominant hand[46]. EMG recordings of Oldowan stone toolmaking further suggest that the muscles stabilizing the thumb (*Flexor pollicis brevis* and *Adductor pollicis transverse*) and little finger (*Flexor digiti minimi* and *Abductor digiti minimi*) may be particularly important in the case of Paleolithic toolmaking [25]. The thumb is of special interest in this context, first because the biomechanics and neuronal control of the human thumb are relatively independent from the other fingers[47], and second because the anatomical relationship of the thumb to the other fingers appears to be derived in humans compared to other apes [48]. Our results show that the variability of the thumb joint configurations during toolmaking (Figure 3, top row) were among the largest of all fingers and thus accounted for much of the manipulative complexity of both Oldowan and Acheulean core grasps. Current fossil and comparative evidence suggests that a reduction in the lengths of the fingers compared to the thumb and an increase in the robusticity of the thumb, both of which would facilitate stable power grasps, occurred relatively early in hominin evolution, prior to or during Oldowan times [48]. Modern apes without these adaptations appear to have difficulty stabilizing stone cores for hand-held percussion, and may adopt alternative strategies such as throwing, bracing against the torso, or even grasping with a foot rather than a hand [49].

While the evolution of motor control for stable hand configurations (stiffness control) is virtually unknown, we suggest that the observed equal manipulative complexity of both Oldowan and Acheulean toolmaking may be due to a common neuronal motor control strategy by which the required mechanical stability of the core is controlled during striking. Thus, skilled toolmaking may have required the evolution and refinement of stiffness control for the hand-arm system by the Early Stone Age, to support tool production.

Previous work has shown that components of variation in human grasp coordination patterns are organized along a gradient from lower to higher finger individuation, with lower order components reflecting coordinated opening and closing of the whole hand and higher order components reflecting fine adjustments of hand shape [50]. As expected, these higher order components distinguish stone toolmaking from even a relatively complex manipulative control task (box stacking: Figure 6D). The fact that they do not distinguish Oldowan from Acheulean toolmaking indicates that differences between these technologies instead lie at a superordinate level of behavioral organization. This higher-level organization allowed the expert subject to achieve different results and to adapt to highly variable object conditions using a comparable array of subordinate grasp synergies.

The adaptive complexity of grasp regulation in Paleolithic toolmaking invites comparison with the complex coordination of articulators (vocal cords, tongue, palate, lips) in speech [51], which must also form flexible synergies in the face of environmental perturbations [52]. This addresses a potential dis-analogy between speech control and manual manipulation, in that the latter might be seen as inherently less ‘arbitrary’ and more environmentally determined [cf. 53]. Current results show that skilled toolmakers are able to impose dynamically stable structure in their manipulative behaviors, despite substantial environmental and task-related variability. In combination with evidence of neural overlap between tool use and phonetic processing in left ventral premotor cortex [5], [54], this supports the argument [34] that Oldowan toolmaking may have provided a ‘preadaptive’ foundation for the enhanced cortical control of vocalization emphasized in many hypotheses of language evolution [37], [51], [55], [56], [57].

Increased right hemisphere activation during Late Acheulean toolmaking (Figure 1D) indicates additional demands for the cognitive control of action in this more complex technology, including a functional/anatomic overlap with discourse-level language processing. Because the structural diversity and complexity of left-hand grips used during Oldowan and Acheulean toolmaking are indistinguishable, these increases in right hemisphere activation cannot be attributed to increases in the basic complexity of contralateral grasp regulation and must instead be attributed to recruitment of distinctive right hemisphere functions. Hypothetically, these include task-set switching and inhibition of contextually inappropriate actions [23] in right inferior frontal cortex (BA45), and the regulation of complex action sequences in right parietal cortex [39], [58]. These functions are consistent with the distinctive behavioral organization of Acheulean toolmaking, which involves switching between different subordinate task-sets in pursuit of superordinate goals to an extent that Oldowan toolmaking does not [6], [34]. For example, properly thinning a Late Acheulean handaxe often requires the toolmaker to stop and prepare edges and surfaces prior to an intended flake removal. This ‘platform preparation’ is accomplished through the small-scale chipping and/or abrasion of edges to alter their sharpness, bevel, and placement relative to the midline [59] and involves its own set of subordinate task goals, operations and tools. Insofar as processing of linguistic context and prosody involves similar demands for the integration of hierarchically structured information over time in the right hemisphere [19], the anatomical overlap of Late Acheulean toolmaking and right hemisphere linguistic processing may reflect the flexible “mapping” of diverse overt behaviors onto shared functional substrates in the brain [37]. This implies that: 1) selection acting on either language or toolmaking abilities could have indirectly favored elaboration of neural substrates important for the other, and 2) archaeological evidence of Paleolithic toolmaking can provide evidence for the presence of cognitive capacities also important to the modern human faculty for language.

## Materials and Methods

### Data Acquisition and Manipulative Complexity Analysis

Hand and arm movements were recorded at a rate of 240 Hz using a Polhemus Liberty electromagnetic tracking system (POLHEMUS, Colchester (VT)). The measurement markers of this system recorded horizontal, vertical and depth position of the marker in a reference coordinate system, as well as the rotational degrees of freedom (yaw, pitch and roll). The system was pre-calibrated to within 3 centimeters precision over the large workspace volume (a cube of about 1.5 by 1.5 by 1.5 meters). Two measurement markers were mounted on a pair of leather gardening gloves. Each marker was fixed on the back of each gardening glove about 2 cm proximal from the knuckle of the middle finger. Gardening gloves were required here to protect the data glove from shear forces and dust/debris during the tool production process. It must be considered that the gardening gloves could possibly have constrained hand movements during the experiment. However, it is not uncommon for such gloves to be worn by experimental stone toolmakers for protection. In this experiment, the gloves did not interfere with the successful production of the intended tools on all eight trials (3 Oldowan, 5 Acheulean), showing that at least the technologically required mobility was present.

The left thigh was used as a support platform for the knapping process and to measure any potentially relevant movements, a third measurement marker was positioned on the kneecap of the left leg. A fourth marker was placed on the back of the tool-maker, at the intersection between the spinal cord and the line connecting the two shoulder joints, so as to account for changes in upper body posture. The core was held and stabilized by the toolmaker's left hand. To record the left hand's grip on the core an 18-sensor CyberGlove I data glove (Cyberglove Systems, San Jose (CA)) was worn underneath the gardening glove (see also Figure 2). The data glove, made of thin cloth with embedded resistive sensors, measures the degrees of freedom of the joints in the hand. Glove sensors were polled at a rate of 80 Hz with a resolution of 8 bits (256 different values) per sensor and calibrated to a splint (see also Figure 3). Glove and marker data stream were resampled to 150 Hz, combined into a single consistent data stream, time-stamped accordingly, and stored for offline analysis. All analysis was conducted with MatLab (MathWorks, Natick (MA)). The full glove time series data set contained between 101,424 and 133,110 data points for the 5 Acheulean toolmaking trials and between 20,840 and 33,486 data points for the 3 Oldowan trials. Automatic annotation tagged about 1.8 to 3.5% of the Acheulean data points, and 4.3% to 6.6% of Oldowan data points, as stable hand configurations. Because all toolmaking data were collected from one of the researchers (the 4^th^ author, an expert stone tool-maker with >40 years of experience) ethics approval and informed consent were not sought.

The dimensionality of hand movements was examined by means of Principal Component Analysis of the joint angles after processing the data, similar to [32], [60], [61]. In our study the data for both the Acheulean and Oldowan sequences was near Gaussian distributed for all joints recorded (Figure 3). This made our dataset ideally suited for Principal Component Analysis. Analysis was performed on the relevant portions of the glove data time series, which was normalized prior to computing the covariance matrix, as our basis for PCA. The event-driven PCA analysis was based on manually tagged or automatically tagged regions of the time series, while the full analysis was based on the complete time series. In contrast to a previous study [32] which considered joint movements (joint velocities), we analyzed angular joint positions (joint angles), as we were interested in the complexity and variability of adaptive hand configurations rather than patterns of movement. We note, that while we applied here PCA as algorithm to calculate our complexity measure, we do not actually reduce the dimensionality of the data, but use all dimensions of the data. Although the hand joint statistics for our actions (Figure 3) are indeed Gaussian distributed, our method would work irrespective of the actual empirical data distribution, as we simply use the amount of variance explained by each principal component (and compute these for all dimensions) as characteristic to measure and distinguish manipulative complexities.

### Calibration

The following two-step calibration was used. First, before each trial the toolmaker placed his hand palm down against a flat surface, with the four fingers parallel and the thumb aligned against the side of the palm (without pressing into the gardening glove's resistance). A reading was then taken from the glove and this served as the zero point for the joint angles in the subsequent recording session. Then, a mechanical splint was placed between the finger joints. Note, however that due to the protective nature of the gardening glove a calibration of the joint sensors to the theoretical maximum precision of <1 degree was difficult, and we operated with about 3 degree resolution – which is within the linear response regime as specified by the manufacturer. After this calibration the toolmaker curled his hand up into a fist. This procedure was performed at the beginning of each trial, after which toolmaking started within 10–30 seconds. In addition, each toolmaking trial was video recorded at 30 Hz using an iSight digital camera (Apple, Cupertino (CA)). A total of 5 Acheulean handaxes and 3 sets of Oldowan stone flakes were produced.

### Manual annotation and automated annotation of data

The focus of the present study was to investigate the complexity of stable core grips. From our own toolmaking and biomechanical experience, the stability and stiffness of the grip was expected to be highest around the time when the hammerstone was going to strike the core. Thus, we manually annotated the video of Acheulean and Oldowan tool making sequences at subsecond precision with the help of ethogram-production software (Etholog, [62]). The annotation recorded seven event types, including two forms of percussion named “percussion”(a hammer strike-like motion) and “light percussion”(a more chiseling-like motion). This very laborious annotation process was only carried out for one Acheulean and one Oldowan sequence. To complement this data set, we developed a simple automated method for detecting these two percussion events. Manual inspection of the position marker data and the glove data synchronized to the video data (Figure 4), produced a straightforward criterion: common to all tool production sequences were characteristic spikes in the velocity plots of the tool hand, of 0.7–2 meters/second for “percussion” and 0.5–1.2 meters/second for “light percussion” events. Thus for all recorded tool making sequences we extracted the glove data when the tool hand moved faster than 0.5 meters/second.

#### Control experiments

Control experiments in two naturalistic tasks (small object sorting and box stacking) were carried out with the same system as was used for the toolmaking experiments. For small object sorting, small (nut-sized) complexly shaped plastic objects (cable carriers: Bosch-Rexroth, Part No. 3842526564) that we refer to here as “widgets” were filled into a container. For box stacking, we used Styrofoam packaging containers (17 cm W ×10 cm H ×19 cm D), with one open side.

Widget sorting task (Figure 5C): Individual widgets were picked out with the left hand from a central container and placed alternately in two containers to the left and right. The size and shape of the widgets typically resulted in 4-finger grip for pick-up and placement. After emptying the central container, objects were picked up individually and alternately from the two adjacent containers and placed back into the central container. This procedure was repeated 3 times.

Box stacking task (Figure 5D): Three Styrofoam boxes were repeatedly precisely stacked upon each other (using the left hand only) and then unstacked. The boxes were normally grasped by the top edge and grip remained effectively constant throughout manipulation, resulting in mainly translatory arm movements and rotation of the wrist. Due to the nature of our two control tasks we could not use limb tracking as a straightforward measure of behavioral actions of the hand during these tasks, because grip phases overlapped broadly with considerable voluntary motion of the hand. Therefore, we analyzed the complete data glove time series of these behaviors and compared these to the complete time series in the toolmaking tasks.
